# Heparin-Induced Thrombocytopenia in a Patient with Essential Thrombocythemia: A Case Based Update

**DOI:** 10.1155/2015/985253

**Published:** 2015-10-22

**Authors:** Edva Noel, Naeem Abbas, Yevegeniy Skaradinskiy, Zwi Schreiber

**Affiliations:** ^1^Department of Medicine, Bronx Lebanon Hospital Center, 1650 Selwyn Avenue, Suite No. 15H, Bronx, NY 10457, USA; ^2^Department of Gastroenterology, Bronx Lebanon Hospital Center, 1650 Selwyn Avenue, Suite No. 10C, Bronx, NY 10457, USA; ^3^Department of Hematology, Bronx Lebanon Hospital Center, 1650 Selwyn Avenue, Suite No. 2C, Bronx, NY 10457, USA

## Abstract

Vascular thrombosis is a common clinical feature of both essential thrombocythemia (ET) and heparin-induced thrombocytopenia (HIT). The development of HIT in a patient with ET is rare and underrecognized. We report the case of a 77-year-old woman with preexisting ET, who was admitted with acute coronary syndrome, and IV heparin was started. She was exposed to unfractionated heparin (UFH) 5 days prior to this admission. Decrease in platelet count was noted, and HIT panel was sent. Heparin was discontinued. Patient developed atrial fibrillation, and Dabigatran was started. On day three, patient also developed multiple tiny cerebral infarctions and acute right popliteal DVT. On day ten of admission, HIT panel was positive, and Dabigatran was changed to Lepirudin. Two days later, Lepirudin was also discontinued because patient developed pseudoaneurysm on the right common femoral artery at the site of cardiac catheterization access. A progressive increase in the platelet count was noted after discontinuing heparin. Physicians should be aware of the coexistence of HIT and ET, accompanied challenges of the prompt diagnosis, and initiation of appropriate treatment.

## 1. Introduction

Essential thrombocythemia (ET) is a myeloproliferative neoplasm characterized by an elevated platelet count greater than 450 × 10^9^/UL, in the presence of acquired pathogenic mutation such as JAK2 or MPL genes and when other causes of reactive thrombocytosis or other myeloid malignancies such as polycythemia vera, primary myeloid fibrosis, chronic myeloid leukemia, or myelodysplastic syndrome have been excluded. One of its most common complications is vascular thrombosis. Heparin-induced thrombocytopenia (HIT) is an immune-mediated disorder which also leads to arterial and venous thrombosis (HIT is an immune-mediated disorder that typically occurs 4–10 days after exposure to heparin and has life and limb-threatening thrombotic complications. In general medical practice, the term HIT refers to type 2 HIT). One of its criteria is a decrease in the platelet count greater than 50% from baseline. We report the case of a patient with preexisting ET, who was exposed to unfractionated heparin (UFH) prior to this admission and who developed acute coronary syndrome, cerebral infarction, and deep venous thrombosis (DVT) of the lower extremity on this admission.

## 2. Case Report

A 77-year-old Hispanic woman was admitted to our hospital with the vague complaints of “not feeling well.” The patient was diagnosed with ET and positive JAK 2 mutation seven years ago with a platelet count stable at 750 × 10^9^/UL. She has been treated with aspirin only. There was no documented history of bleeding, thrombosis, or other symptoms. Her other medical comorbid included benign essential hypertension, heartburn, osteoarthritis, and depressive disorder. Over an eighteen-day period the patient has had two consecutive six-day duration hospitalizations, for chest pain and fall, respectively. She was lastly discharged five days prior to this new admission. In those 2 previous admissions she received subcutaneous heparin for deep venous thrombosis (DVT) prophylaxis. At the time of the current admission her vitals were unremarkable, and so was the rest of the physical examination.

The first set of blood works revealed the following: hemoglobin 14.9 g/dL (reference range 12–15.5 g/dL); white blood cell count 31.6 × 10^3^/UL (reference range 4.8–10.8/UL) with 92.6% neutrophils; platelet count 285 × 10^9^/UL (reference range 150–400/UL); BUN 71 (reference range 6–20 mg/dL); creatinine 3.6 mg/dL (reference range 0.5–1.5 mg/dL); ALT 8624 mg/dL (reference range 5–40 mg/dL); AST 13824 mg/dL (reference range 9–36 mg/dL); troponin T 0.952 ng/mL (reference <0.100 ng/mL); total creatinine kinase 193 Unit/L (reference range 20–200 Unit/L); creatinine kinase MB 4.77 ng/mL (reference range <0.500 ng/mL).

During this admission, serum cardiac markers were elevated, and electrocardiographic changes were nonspecific. Intravenous heparin was started for (non-ST-elevation myocardial infarction) NSTEMI. Further record review showed that the platelet count decreased by more than 50% (platelet count 285 × 10^9^/UL on CBC at the time of the current admission compared to the platelet count of 750 × 10^9^/UL at the time of the previous discharge 5 days ago). Furthermore, the platelet count reached a nadir of 216 × 10^9^/UL. UFH was discontinued, but direct thrombin inhibitor was not started because suspicion for HIT was deemed to be low. An echocardiogram showed atrial fibrillation, low normal left ventricular ejection fraction (EF). Dabigatran was started for atrial fibrillation. On the second day of admission the kidney function improved on IV hydration, and the patient underwent cardiac catheterization. The study showed patent coronary arteries. In the late evening of day two, the patient became acutely confused and agitated. Computer tomography scan (CT) and magnetic resonance imaging (MRI) of the brain showed multiple tiny infarcts in the right cerebellum, right occipital and left frontal lobes. An ultrasound of the lower extremities showed acute right popliteal vein thrombosis. An inferior vena cava (IVC) filter was placed. On day five of hospitalization heparin platelet factor 4 (HPF4) antibody test came back negative. Positive test does not necessarily indicate the presence of thrombosis or thrombocytopenia, since only a minority of PFH4/heparin antibodies activate the platelets. Further confirmatory test (Serotonin Release Assay) C^14^-SRA was sent on day six of hospitalization which came back positive. Whilst awaiting for the gold standard test of C^14^-SRA, patient was continued on Dabigatran for new onset atrial fibrillation which, after the confirmed diagnosis of HIT, was discontinued and Lepirudin started. On twelfth day, the patient complained of discomfort at the right groin. Examination revealed ecchymosis with a pulsatile nontender mass at the right groin. Duplex of the right groin showed pseudoaneurysm of the right common femoral artery; Lepirudin was discontinued. On thirteenth day, the patient underwent successful obliteration of the pseudoaneurysm by in situ thrombin injection. The patient was observed in the hospital for two more days. Subsequently no bleeding or thrombotic event occurred. After discontinuing heparin, platelet count progressively increased to reach 815 × 10^9^/UL at the time of discharge, Table 1. The patient was discharged only on aspirin because she did not opt to continue on anticoagulation owing to bleeding risk.

## 3. Discussion

Both prevalence and incidence of HIT are reported <1% with very high mortality (1 in 3), and nadir platelet count was the only factor determining this dreadful outcome [1]. A retrospective study from CATCH registry revealed that HIT was not suspected in most of the patients despite a significant decline in platelet counts. When it was suspected heparin was not discontinued and direct thrombin inhibitor was not initiated in timely manner [2]. That suggests the underrecognition of this common clinical entity by the physician which bears very severe consequences of 33% risk of thromboembolic events.

Platelet factor 4 (PF4) level can predict the development of HIT and increase the thrombogenic risk. High risk factors include older age, female gender, cardiovascular disease, or surgical manipulation [3]. Though other causes of thrombocytopenia are more common in in-patient setting, such as thrombocytopenia due to sepsis or dilutional thrombocytopenia, HIT is an underrecognized condition and 4T score should be used to predict the pretest probability of HIT [4]. Hypercoagulable states such as DVT, stroke, acute myocardial infarction, and postoperative states especially orthopedic surgery confer higher risk of HIT [5]. Prolonged exposure (>1 week) and early reexposure (within 100 days) of IV heparin are well-established factors for the development of HIT and HITT (heparin-induced thrombocytopenia with thrombocytosis). The use of subcutaneous UFH in hospital setting for prophylactic purposes results in lower incidence of HIT (0.8%), but the risk of thrombotic events is similar to HIT following IV heparin therapy [6]. Warkentin raised concerns over the possibility of overdiagnosis of HIT in ICU setting, based on thrombocytopenia, and potential of serious consequences of initiation of thrombin inhibitors in these patients who already harbor higher risk of bleeding because of coagulopathy, mechanical ventilation, and renal or hepatic impairment. It was suggested that Dalteparin can be used as prophylaxis for DVT and (pulmonary embolism) PE and has very low risk of HIT (0.6%) as compared to UFH [7]. HIT can have extensive thrombosis in unusual locations such as heart. Despite the high sensitivity and specificity of HPF4 and SRA (ELISA test of HPF4 antibody: sensitivity >90%, specificity 50–93%; Serotonin Release Assay: sensitivity 90–98%, specificity 80–97%), the diagnostic test can be negative in the beginning of symptoms and should be repeated if clinical suspicion is high [8].

Essential thrombocythemia confers higher risk of thrombosis. Development of HIT in patients with ET has been reported and poses diagnostic challenge because despite more than 50% decline in platelets count, absolute count of platelets may be within reference laboratory range. With a negative predictive value of 99% the “4 T's rule” has been used in establishing a level of clinical suspicion and ruling out HIT. However, in patients with ET, HIT can occur at a platelet count which would be considered normal in the general population. The patients with ET usually exhibit severe decline in platelet counts, in some cases reaching up to 98%. We believe that the percentage of the decrease in the platelet count is a more reliable parameter than the absolute platelet count to raise suspicion for HIT in ET patients [9].

Increased level of PF4 in patients with ET has been postulated to the development of HIT [10]. Risch et al. described the first case of HIT developing after heparin therapy in patient with ET who developed recurrent stroke at nadir platelet count value of 633,000/mm^3^ [11]. Since then 9 more cases have been reported (Table 2).

ET can be asymptomatic, and bone marrow biopsy as workup for thrombocytopenia (HIT) can reveal an unusual combination of ET and HIT [12]. To avoid the delay in diagnosis and its attendant complication, delta change in platelet count rather than the absolute value should be considered and evaluated whilst considering the diagnosis of HIT in the setting of ET [13].

Recommendations from the CHEST in 2012 include monitoring of platelets every 2-3 days from day 4 to day 14 of heparin therapy if risk of HIT is >1% and platelets to be transfused only if there is active bleeding or invasive procedure is being performed. Vitamin K antagonist (VKA) should be started only when the platelet count has risen to significant level, usually more than 150,000/UL. VKA should be started at lower dose and overlapped with other nonheparin anticoagulants such as Argatroban, Lepirudin, or Danaparoid for at least five days [14].

Our patient developed HIT at a platelet count of 750 × 10^9^/UL. Even though HIT was suspected, the level of suspicion was deemed to be low. As platelet count dropped further after starting IV heparin, the latest one was discontinued, and appropriate laboratory tests were sent to confirm the diagnosis, recommended therapeutic interventions in the form of nonheparin anticoagulants such as Argatroban, Lepirudin, or Danaparoid were started only after confirming the diagnosis. This patient was started on Dabigatran for atrial fibrillation. Patient developed multiple tiny cerebral infarcts; atrial fibrillation might have contributed to them, but the patient also developed acute DVT of right popliteal vein. This extensive thrombosis at multiple anatomic locations was due to HIT as confirmed by laboratory data and gradual increase in platelet count that was noted after discontinuation of IV heparin. This case also had diagnostic challenge as patient developed thromboembolic phenomenon when the platelet count was within laboratory reference range. This case also underscores the importance of starting the recommended nonheparin anticoagulants, if the clinical and pretest probability for HIT is high.

## 4. Conclusion

HIT is a complication of the use of heparin in clinical settings. Clinicians have to be alert that preexisting ET may hide the occurrence of HIT. Therefore, we recommend that, in a patient with ET who was exposed to heparin, a decrease in the platelet count greater than 50% from the base line along with a high clinical suspicion should prompt the initiation of the appropriate management.

## Figures and Tables

**Table 1:** 

	5 days earlier	Admission day	Day 2	Day 4	Day 5	Day 6	Day 10	Day 14	Discharge day
Platelets × 10^9^/UL	750	285	216	230	239	263	583	870	815
Events	—	NSTEMI	Stroke, A. fib., and DVT	—	—	—	—	Pseudoaneurysm of right femoral artery	—
Heparin	—	Started	Discontinued	—	—	—	—	—	—
Dabigatran	—	—	Started	Continued	Continued	Continued	Discontinued	—	—
PF4 IgG Ab	—	—	Test sent	Positive result	—	—	—	—	—
C^14^-SRA	—	—	—	—	—	Test sent	Positive result	—	—
Lepirudin	—	—	—	—	—	—	Started	Discontinued	—

**Table 2:** 

Case number	Hematologic disorder	Age/sex	Disease treated with heparin	Initial platelet count × 10^9^/UL	Platelet nadir × 10^9^/UL	New thrombotic event	Coagulation disorder/organ failure	Fibrinogen degradation product	Author/year
1	ET	52/F	Cranial sinus thrombosis	1008	109	None	None	No evidence of DIC	Walther et al. (1996) [15]

2	ET	57/M	Transient ischemic attack	1235	633	None	None	Not described	Risch et al. (2000) [11]

3	ET	78/F	Subclavian and axillary thrombosis	965	194	None	None	Not described	Houston (2000) [10]

4	ET	45/F	Mesenteric and portal vein thrombosis	840	110	Femoral vein thrombosis and subclavian venous thrombosis	None	Not described	Spectre et al. (2008) [16]

5	ET	71/F	Spleen infarction	640	383	Heparin-induced skin necrosis	None	Not described	Spectre et al. (2008) [16]

6	ET	38/F	Budd-Chiari syndrome	175	78	Pulmonary embolism	Not described	Not described	Randi et al. (2010) [17]

7	ET	66/F	DVT and pulmonary embolism	700	6	Not described	Not described	Not described	Lapecorella et al. (2010) [12]

8	ET	61/F	Cerebral venous thrombosis	517	42	Extension of cerebral venous thrombosis	None	Not described	Richard et al. (2011) [13]

9	ET	67/F	Cerebral infarction	1270	7	Cerebral infarct, femoral artery occlusion, and thrombophlebitis of extremities	Dyspnea, petechiae, and hematuria	Increased by 20–30 *µ*L	Murawaki et al. (2012) [9]

Present case	ET	67/F	NSTEMI	750	216	Cerebral infarct, popliteal vein thrombosis	Acute renal failure	No evidence of DIC	Present
